# A Rare Cause of Recurrent Lower Gastrointestinal Bleed: Colonic Dieulafoy’s Lesion

**DOI:** 10.7759/cureus.20384

**Published:** 2021-12-13

**Authors:** Yvette Achuo-Egbe, Syed Salman H Hashmi, Ahmed Shady, Gulam M Khan

**Affiliations:** 1 Gastroenterology and Hepatology, New York Medical College, Metropolitan Hospital Center, New York, USA; 2 Internal Medicine, New York Medical College, Metropolitan Hospital Center, New York, USA; 3 Gastroenterology, Woodhull Medical Center, Brooklyn, USA

**Keywords:** dieulafoy’s lesion, melena, obscure bleeding, endoscopic intervention, thermocoagulation, hemoclipping, severe anemia, gi bleeding, colon

## Abstract

Dieulafoy’s lesion accounts for 1%-2% of acute gastrointestinal (GI) bleeding cases, and approximately 2% of Dieulafoy’s lesions are present in the colon. We report the case of an 83-year-old female who presented with recurrent gastrointestinal bleeding from colonic Dieulafoy’s lesion located at the hepatic flexure. She initially presented four weeks prior with melena in the setting of Eliquis use for venous thrombosis, coronary artery disease, and end-stage renal disease. Upper endoscopy revealed esophagitis, gastritis, and duodenitis. Diagnostic colonoscopy and video capsule endoscopy both revealed blood in the colon without an identifiable source. During the second admission for recurrent melena with hemoglobin of 3.9 g/dL, Eliquis was discontinued, and the patient was resuscitated with three units of packed red blood cell transfusions. Repeat colonoscopy revealed a pulsating vessel with active oozing located at the hepatic flexure, consistent with a Dieulafoy’s lesion. Hemostatic endoclips and bipolar electrocautery were applied to achieve complete hemostasis. Colonic Dieulafoy’s lesions, albeit rare, should be considered in patients presenting with an acute obscure lower GI bleed. Primary hemostasis can be achieved with several endoscopic modalities including epinephrine, hemoclipping, thermocoagulation, or sclerotherapy.

## Introduction

Dieulafoy’s lesion is a dilated aberrant submucosal artery that erodes the overlying mucosa in the absence of mucosal ulceration. It accounts for 1%-2% of acute gastrointestinal bleeding cases. Over 70% of Dieulafoy’s lesions are typically found in the stomach, and approximately 2% are present in the colon [1,2]. These lesions are extremely rare causes of lower gastrointestinal (GI) bleeding compared to the common etiologies such as diverticulosis, angiodysplasia, and ischemia. We report the case of an elderly patient with recurrent gastrointestinal bleeding requiring multiple endoscopic evaluations to identify etiology as Dieulafoy’s lesion and hemostasis achieved with endoclips and bipolar cautery.

## Case presentation

**Figure 1:**
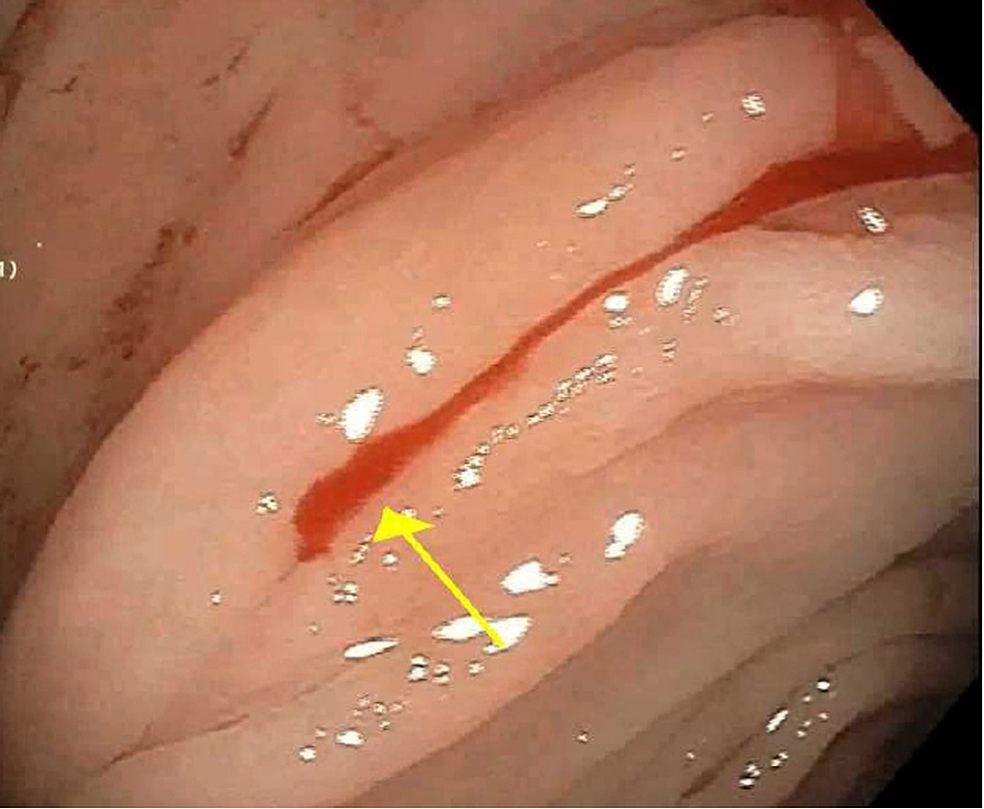
Active bleeding from colonic Dieulafoy’s lesion. The yellow arrow points to the site of the Dieulafoy’s lesion.

**Figure 2:**
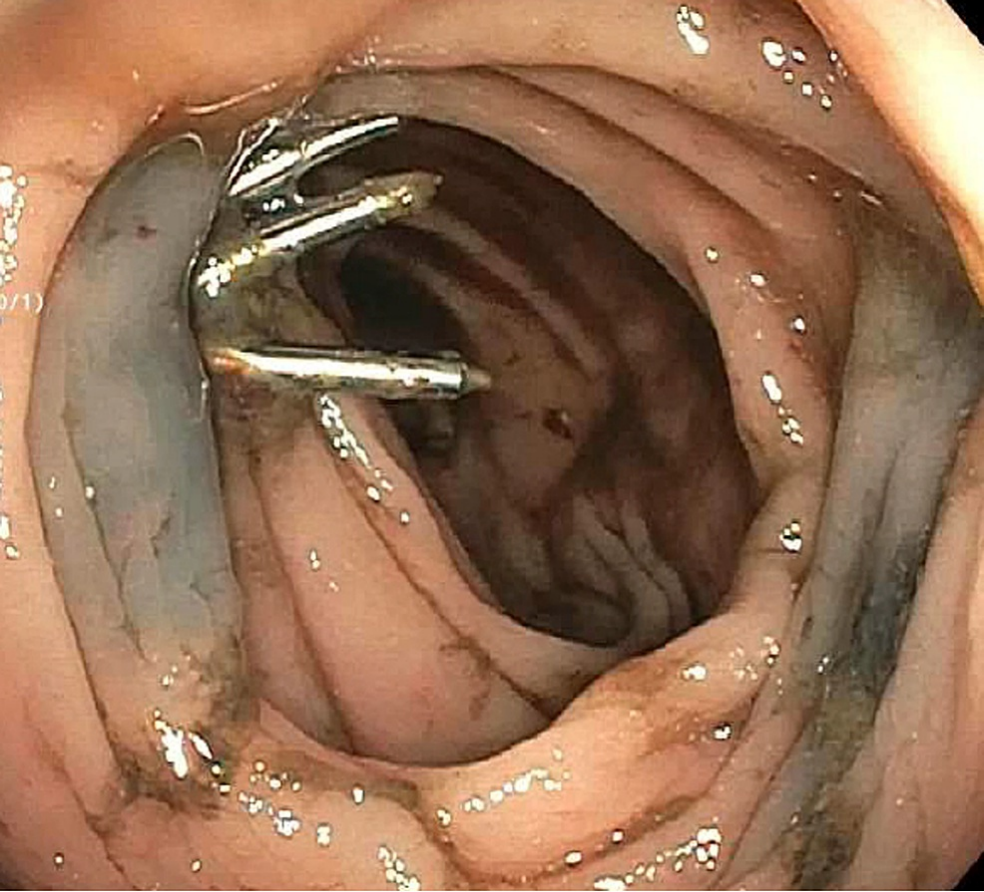
Hemoclips deployed at the site of bleeding with thermal coagulation to achieve hemostasis and area tattooed.

The patient is an 83-year-old female with end-stage renal disease, coronary artery disease, diabetes mellitus, hypertension, internal jugular thrombosis, and arteriovenous fistula thrombosis on Eliquis, with prior admission four weeks ago for intermittent melena and blood loss anemia with hemoglobin of 5 g/dL. Esophagogastroduodenoscopy revealed candida esophagitis, gastritis, and duodenitis. Diagnostic colonoscopy and subsequent video capsule endoscopy both revealed blood in the colon without an identifiable source of bleeding. The patient received packed red blood cell transfusions and was discharged on candida treatment and a proton pump inhibitor. The patient was later hospitalized with a traumatic femur fracture after a fall and recurrent melena. She was hemodynamically stable on admission. Her initial hemoglobin level was 3.9 g/dL. Unenhanced computed tomography revealed no fluid collections in the abdomen. Physical examination revealed pale skin and nontender, nondistended abdomen, and rectal examination showed melena. Eliquis was stopped, and the patient was resuscitated with intravenous crystalloid fluids and three units of packed red blood cell transfusions. Repeat colonoscopy revealed a pulsating vessel with active oozing located at the hepatic flexure, consistent with a Dieulafoy’s lesion (Figure 1). Four hemostatic clips were deployed at the bleeding site, and bipolar electrocautery was applied to achieve complete hemostasis (Figure 2). The lesion was tattooed proximally and on the opposite mucosal wall. The patient remained hemodynamically stable with no recurrent bleeding and stable hemoglobin levels postoperatively. She was discharged home with close follow-up at the gastroenterology clinic.

## Discussion

Dieulafoy’s lesion was first described in 1898 by a French surgeon, Paul George Dieulafoy, as superficial gastric lesions without typical features of gastric ulcers in his paper “Exulceratio simplex: Leçons 1-3” [3,4]. Colonic Dieulafoy’s lesion was reported by Barbier et al. in 1985 as an extreme cause of lower gastrointestinal bleeding, posing diagnostic and therapeutic challenges [2]. Approximately 2% of all Dieulafoy’s lesions are colonic, with several studies having demonstrated incidence throughout the entire colon [2-8].

Dieulafoy’s lesions are characterized by painless bleeding and can present as melena or hematochezia, depending on the location and colonic transit time. This presentation is also similar to colonic bleeding due to angiodysplasia and diverticulosis. The risk of bleeding increases with certain preexisting comorbidities, including cardiovascular disease and hypertension with or without simultaneous use of anticoagulation [2,9].

Bleeding from Dieulafoy’s lesions is typically classified as acute obscure-overt gastrointestinal bleeding with negative initial diagnostic evaluation to identify the source of bleeding. The diagnostic challenges of these lesions could stem from inadequate bowel preparation, obscured visual field from the large volume of blood present, and subtlety and location of the lesions, sometimes buried within large mucosal folds. Diagnostic modalities such as computed tomography angiography, direct mesenteric angiography, and red cell scintigraphy have been utilized in identifying Dieulafoy’s lesions [2].

Primary hemostasis can be achieved with endoscopic intervention, most common including epinephrine injection and hemoclipping, with over 90% success rate [1,10-12]. Studies report that these endoscopic interventions achieve sustained hemostasis for at least six months [2]. Other therapeutic endoscopic modalities that have been less utilized and sometimes in combination include thermal coagulation, endoscopic sclerotherapy, and argon plasma coagulation [2,11]. Rescue therapy utilized during recurrent bleeding from Dieulafoy’s lesions despite endoscopic interventions includes angiographic embolization or surgery [1,2,12], a similar approach for gastric lesions. Angiographic embolization can also be utilized in poor surgical candidates and as part of combination therapy [2,12].

## Conclusions

Colonic Dieulafoy’s lesions should be considered in patients presenting with acute obscure lower gastrointestinal bleed. Localization of the lesion and primary hemostasis are mostly achieved endoscopically with epinephrine, hemoclipping, or in combination with thermal coagulation or endoscopic sclerotherapy.
